# Development of a capillary electrophoresis–mass spectrometry method for the analysis of metformin and its transformation product guanylurea in biota

**DOI:** 10.1007/s00216-020-02759-6

**Published:** 2020-06-22

**Authors:** Sarah Knoll, Stefanie Jacob, Susanna Mieck, Rita Triebskorn, Thomas Braunbeck, Carolin Huhn

**Affiliations:** 1 Institute of Physical and Theoretical Chemistry, University of Tübingen, Auf der Morgenstelle 18, Tübingen, Germany; 2 Animal Physiological Ecology Group, Institute of Evolution and Ecology, University of Tübingen, Auf der Morgenstelle 5, Tübingen, Germany; 3 Aquatic Ecology and Toxicology Group, Centre for Organismal Studies, University of Heidelberg, Im Neuenheimer Feld 504, Heidelberg, Germany

**Keywords:** Pharmaceuticals, Ecotoxicology, Brown trout, Zebrafish, Sample preparation

## Abstract

A method with capillary electrophoresis coupled to mass spectrometry was optimized to determine the uptake of metformin and its metabolite guanylurea by zebrafish (*Danio rerio*) embryos and brown trout (*Salmo trutta* f. *fario*) exposed under laboratory conditions. Metformin was extracted from fish tissues by sonication in methanol, resulting in an absolute recovery of almost 90%. For the extraction of guanylurea from brown trout, solid-phase extraction was implemented with a recovery of 84%. The use of a mixture of methanol and glacial acetic acid as a non-aqueous background electrolyte was vital to achieve robust analysis using a bare fused-silica capillary with an applied voltage of +30 kV. Problems with adsorption associated with an aqueous background electrolyte were eliminated using a non-aqueous background electrolyte made of methanol/acetic acid (97:3) with 25 mM ammonium acetate (for zebrafish embryos) or 100 mM ammonium acetate (for brown trouts), depending on the sample complexity and matrix influences. High resolution and high separation selectivity from matrix components were achieved by optimization of the ammonium acetate concentration in the background electrolyte. An extensive evaluation of matrix effects was conducted with regard to the complex matrices present in the fish samples. They required adapting the background electrolyte to higher concentrations. Applying this method to extracts of zebrafish embryos and brown trout tissue samples, limits of detection for both metformin and guanylurea in zebrafish embryos (12.2 μg/l and 15 μg/l) and brown trout tissues (15 ng/g and 34 ng/g) were in the low μg/l or ng/g range. Finally, metformin and guanylurea could be both quantified for the first time in biota samples from exposure experiments.

**Electronic supplementary material:**

The online version of this article (10.1007/s00216-020-02759-6) contains supplementary material, which is available to authorized users.

## Introduction

Micropollutants like pharmaceuticals are increasingly perceived as a major hazard to aquatic ecosystems worldwide, due to insufficient removal or degradation in wastewater treatment plants [1]. The bioaccumulation of pharmaceuticals in biota, especially the uptake of polar and charged substances, has barely been studied [2, 3]. The present study focuses on the antidiabetic drug metformin, a pharmaceutical with very high polarity (log *D* = − 5.7, pH 7). At environmentally relevant pH, metformin is present as a doubly charged cation (Table 1). For decades, metformin has been used as an effective pharmaceutical in the treatment of type 2 diabetes mellitus [4]. The administered daily dosage (metformin hydrochloride) ranges from 500 to 2500 mg. Metformin is one of the pharmaceuticals with the highest mass production [5], and its use will surely increase further with rising patient numbers [6]. In humans, metformin is not metabolized and thus passes the body unmodified [7]. In wastewater treatment plants, metformin is partially transformed to guanylurea by microbial activity; therefore, large amounts of both compounds are released into the aquatic environment [8, 9]. In surface waters, metformin concentrations range from 0.1 to 1.7 μg/l and those of guanylurea from 0.1 to 25 μg/l [10]. There is limited knowledge on both the bioaccumulation and the biological effects of both substances in aquatic organisms: (eco)toxicological studies showed an LC_50_ value of > 982 mg/l for bluegill sunfish (*Lepomis macrochirus*) and an EC_50_ value of 130 mg/l for *Daphnia magna* [11]. Studies with brown trout (*Salmo trutta* f. *fario*) larvae revealed changes of the liver glycogen, reduced body weight, and an influence on the gut microbiome already at environmentally relevant concentrations of 1 μg/l [12], whereas brown trout larvae, exposed to guanylurea at concentrations of 1–100 μg/l, did not show effects [13]. Another ecotoxicological study with both compounds and big ramshorn snails (*Planorbarius corneus*) only showed effects at concentrations 10,000 times higher than environmentally relevant [14].

**Table 1:**
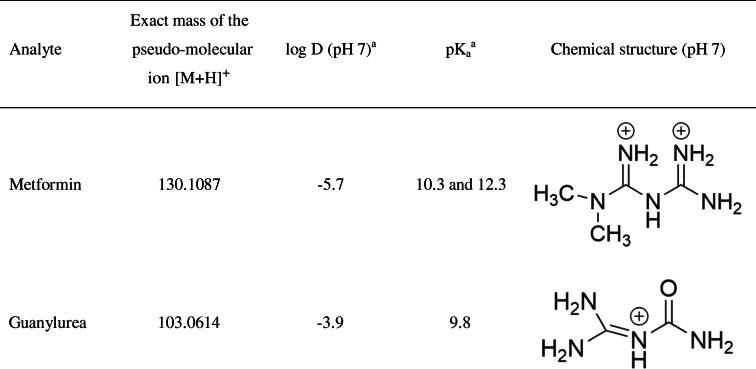
Chemical structures of metformin and guanylurea with exact mass of the pseudo-molecular ion [M+H]^+^, polarity (log *D*; pH 7), and p*K*_a_

^a^Provided by chemicalize.org

Reversed phase liquid chromatography (RPLC) coupled with mass spectrometry (MS) is most often used for the analysis of micropollutants but has limitations regarding the analysis of very polar substances such as metformin and guanylurea (rf. Table 1), especially when the salt content of samples is high [15]. Alternative chromatographic modes are hydrophilic interaction liquid chromatography (HILIC) and ion-pair liquid chromatography (IC). Both methods were often applied for the analysis of metformin in human plasma [16–19]. In environmental analysis, HILIC was implemented for the analysis of metformin and guanylurea in sewage, surface, and wastewater [9, 20]. However, both techniques suffer from several drawbacks: For example, IC is prone to long column equilibration times, potential impurities from ion-pair reagents, and limited compatibility with MS [21]. For HILIC, method development is very complex and it is time-consuming to find the optimum chromatographic conditions. Compared to RP methods, the robustness is low, leading to a limited repeatability [20]. HILIC also often suffers from matrix problems, especially in samples containing high concentrations of salts. Moreover, the column durability is affected, due to the higher matrix content of complex samples. A major drawback for water samples is the need to dilute the sample to reach a solution with ca. 80% organic solvent.

Capillary electrophoresis (CE) is an interesting alternative for the analysis of ionizable compounds and especially permanently charged compounds also in environmental applications [22]. CE was used to quantify metformin in tablets [23, 24], plasma [25, 26], and serum [27, 28] and rarely in other biofluids like urine [28]. CE is often used with UV detection at wavelengths below 230 nm (as low as 195 nm) [22] sometimes with capacitively coupled contactless conductivity detection (C^4^D) and once with electrochemiluminescence (ECL) detection [29]. We are aware of only one publication on CE-MS analysis of metformin [27]. Ben-Hander et al. [24] gave a summary on all these CE methods. Limits of detection (LOD) covered a very broad range. CE-UV or CE-C^4^D often reached LODs in the mg/L range, sometimes upper μg/l range, sufficient for metformin analysis in tablets and often also in plasma (therapeutic concentrations are 0.1–1 mg/l [29]). Only with MS or ECL detection or with dedicated sample preparation, lower values were reached [24]. We are not aware of any publication dealing with metformin analysis in biota. Overall, CE-MS studies for aquatic organisms are scarce. There are some studies using CE for the analysis of shellfish toxins [30, 31] and pharmaceuticals such as the polar tetracyclines [32, 33] and quinolones [34] in aquatic organisms.

Regarding sample preparation for metformin analysis in various matrices (serum, environmental samples), most frequently solid-phase extraction (SPE) [8, 26] or protein precipitation [16, 27] was used. Due to the high concentrations in tablets and plasma samples, a direct injection (with dilution only) was often possible for both LC and CE with minor or no matrix effects observed (see summary by Ben-Hander et al. [24]).

In this study, we developed a novel analytical method based on CE-MS to determine the tissue concentration of metformin and guanylurea at a level of a few nanograms/gram in zebrafish embryos and brown trout, originating from exposure experiments. To the best of our knowledge, this is the first application of CE-MS for environmental biota analysis. The advantages of the method are good LODs despite the low loadability of CE capillaries (i.e., a few nanoliters), the speed of analysis (total separation time under 12 min), the low solvent consumption, and the small sample preparation effort to quantify the target analytes in different fish matrices. We here show that with a non-aqueous background electrolyte (BGE), high specificity, selectivity, precision, and matrix tolerance can be reached with a LOD in the low μg/l or ng/g range for both compounds. The small sample requirements of only a few nanoliters for CE also enabled the analysis of metformin in specific organs (liver, kidney, intestines, gill, and muscle) from homogenates of five juvenile brown trouts to determine the distribution of metformin in the organism.

## Material and methods

### Chemicals

Methanol (MeOH) hypergrade LC-MS (Chromasolv), water hypergrade LC-MS (Chromasolv), acetonitrile (ACN) (LC-MS grade), isopropanol (LC-MS grade), and formic acid (98%) were purchased from Sigma-Aldrich (Steinheim, Germany). The pharmaceutical standards metformin hydrochloride and guanylurea sulfate were supplied by Sigma-Aldrich, whereas deuterated metformin-d6 was purchased from Toronto Research Chemicals (North York, Canada). An isotopically labeled standard for guanylurea was not commercially available. Ammonium acetate (NH_4_OAc) (98%) and glacial acetic acid (HOAc) (100%) were obtained from Merck (Darmstadt, Germany). Cartridges for SPE (Strata-X-CW, 30 mg) were supplied by Phenomenex (Aschaffenburg, Germany). A 3-mm PTFE syringe filter (0.45 μm) was supplied by Macherey-Nagel (Düren, Germany). Individual stock solutions of metformin and guanylurea with a concentration of 1 g/l were prepared in LC-MS grade water. The stock solution of metformin-d6 with a concentration of 1 g/l was prepared in MeOH (hypergrade LC-MS). All working solutions of the standards and samples for direct injection were prepared in MeOH containing 10% BGE using the 25 mM NH_4_OAc in MeOH:HOAc (97:3) to avoid current breakdown and band broadening due to field amplification [35, 36]. Stock and working solutions were stored at − 20 °C before use.

### Instrumentation and CE-TOF-MS procedures

All analyses were performed using an Agilent CE 7100 (Agilent Technologies, Waldbronn, Germany) interfaced to an Agilent 6550 iFunnel Q-TOF MS (Agilent Technologies, Santa Clara, USA) with an electrospray ionization (ESI) source assisted by a sheath liquid interface (Agilent Technologies, Waldbronn, Germany). The composition of the sheath liquid was isopropanol/water (1:1, v/v) with 0.01% formic acid. MeOH was also tested as sheath liquid solvent; however, it improved neither MS signal intensity nor spray stability, but background signals were elevated. A higher concentration of formic acid (0.1%) was tested, but signal intensity decreased. The sheath liquid was delivered by a 1260 isocratic pump (Agilent Technologies, Waldbronn, Germany) at a flow rate of 5 μl/min using a flow splitter (split ratio 1:100). The nebulizer pressure was set to 6 psi and the drying gas flow rate to 11 l/min. A fragmentor voltage of 380 V, a capillary voltage of − 4000 V, a skimmer voltage of 65 V, and an octopole voltage of 750 V were used. The mass range was set to m/z 50–1700, and the data acquisition rate was 2 spectra/s. For internal calibration, purine, HP0321, and HP0921 (Agilent Technologies, Waldbronn, Germany) were added to the sheath liquid. Data analysis was accomplished using MassHunter software (Agilent Technologies, Waldbronn, Germany).

The CE separations were carried out using a bare fused-silica capillary (length 80 cm, 50 μm i.d.; Polymicro Technologies, Phoenix, Arizona). For standard solutions and zebrafish embryo extracts, the optimized condition of the BGE was a mixture of 25 mM NH_4_OAc in MeOH:HOAc (97:3). For brown trout extracts, the BGE was adapted to 100 mM NH_4_OAc in MeOH:HOAc (97:3). Samples were injected hydrodynamically by applying a pressure of 100 mbar for 10 s. New capillaries were conditioned with BGE for 15 min and before each run for 5 min. Activation of the capillaries was not necessary, since we did not observe any improvements in precision. The CE capillary was kept at 25 °C during CE runs, and a voltage of + 30 kV was applied. The capillary was kept in BGE upon storage.

## Uptake experiments

### Exposure of zebrafish embryos

The fish embryo toxicity tests were performed according to OECD test guideline 236 [37]. Fertilized zebrafish (*Danio rerio*) were collected in a stage ranging from 4 to 32 cells and exposed to six concentrations of metformin (0 g/l, 0.1 g/l, 0.5 g/l, 1.0 g/l, and 2.0 g/l). The test solutions were replaced by freshly prepared medium on a daily basis. After 96 h post fertilization, the embryos were washed three times in deionized water, euthanized with an overdose of tricaine (400 mg/l), frozen in liquid nitrogen, and stored as pools of five embryos per concentration at − 80 °C until analysis. Further information regarding the embryo toxicity tests is provided in the Electronic Supplementary Material (ESM).

### Exposure of brown trout larvae

In this work, the experimental procedure and the results are described exemplarily for metformin. Detailed information about the exposure experiment with guanylurea and brown trout larvae is given elsewhere [13]. The exposure experiment with metformin and brown trout (*Salmo trutta* f. *fario*) larvae was conducted as described by Jacob et al. [12]. In brief, brown trout in eyed-ova stage (age, 48 days post fertilization) were exposed in triplicate to five different treatments of metformin (0 μg/l, 1 μg/l, 10 μg/l, 100 μg/l, and 1000 μg/l) at 7 °C and 11 °C in climate chambers. The exposure took place in glass aquaria in a semi-static system with 28 test organisms per aquarium. The experiment was terminated 8 weeks after yolk sac consumption, corresponding to an exposure duration of 95 days at 11 °C and 108 days at 7 °C. As some organs were used for effect studies [12], 21 samples (from the head (without the gills) to the tail fin, including the kidney and muscle, but not the intestines or liver) per treatment were taken and immediately frozen in liquid nitrogen for chemical analyses.

### Exposure of juvenile brown trout

Brown trout at an age of 1 year were exposed to four concentrations of metformin (0 μg/l, 1 μg/l, 10 μg/l, and 1000 μg/l) at 7 °C in a climate chamber. The exposure experiment was conducted in a semi-static system using glass aquaria with five test organisms per 20-l aerated test medium. Twice a week, 50% of the exposure medium was exchanged by freshly prepared medium. Aerated and filtered tap water (iron filter, active charcoal filter, particle filter) was used for the preparation of the medium. Brown trout were fed daily with commercial trout food (INICIO plus 0.8 mm from BioMar Denmark). During the water exchange process, excess food and feces were removed. After 23 days of exposure, fish were euthanized with an overdose of MS 222 (1 g/l buffered in NaHCO_3_) and subsequent severance of the spine. Samples for chemical analysis of the liver, kidney, intestines, gills, and muscle were taken and immediately frozen in liquid nitrogen.

## Preparation of matrix-matched standards

One hundred milligrams of homogenized blank fish tissue were transferred to an Eppendorf tube and extracted as described in “Methanolic extraction.” The blank extracts were spiked with standard solution to reach the established calibration range for metformin and guanylurea (Table 3). Prior to CE-MS analysis, the extracts were filtered with a 45-μm PTFE filter.

## Preparation of the fish samples

### Extraction of metformin and guanylurea from zebrafish embryos

For extraction of the target analytes, 450 μl MeOH and 50 μl deuterated internal standard solution (metformin-d6, *c* = 1 mg/l; final concentration in injection solution = 0.1 mg/l) were added to an Eppendorf tube, each containing five zebrafish embryos. The tube was vortexed, and the analytes were extracted under sonication for 15 min. After centrifugation at 13,000*g* for 15 min, the supernatant was collected and diluted with MeOH (LC-MS grade) + 10% BGE (25 mM NH_4_OAc in MeOH:HOAc (97:3)) to reach concentrations compatible with the calibration range established for the analyte. After filtration with 45-μm PTFE filters, the sample was analyzed by CE-MS.

### Extraction of metformin and guanylurea from brown trout

#### Methanolic extraction

Frozen (− 20 °C) brown trout samples (either brown trout larvae from the head without the gills to the tail fin, including the kidney and muscle, but not the liver or intestines and brown trout (1-year-old) muscle or organs) were first homogenized by grinding with a mortar and pestle under liquid nitrogen. A total of 100 mg of the homogenized sample was then transferred to an Eppendorf tube, and 50 μl deuterated internal standard solution (metformin-d6, *c* = 0.5 mg/l; final concentration in injection solution = 0.05 mg/l) and 450 μl MeOH as extraction solvent were added. The tube was vortexed for 30 s, and the analytes were extracted under sonication for 15 min. After centrifugation at 13,000*g* for 15 min, the supernatant was filtered with a 45-μm PTFE filter prior to CE-MS analysis.

#### Solid-phase extraction

One hundred milligrams of the homogenized fish tissue were transferred to an Eppendorf tube, and 50 μl internal standard solution (metformin-d6, *c* = 0.5 mg/l; final concentration in injection solution = 0.3 mg/l) and 1.45 ml water were added, followed by vortexing for 30 s. After centrifugation for 15 min, 1 ml of the supernatant was transferred to the SPE cartridge (Strata-X-CW). Prior to loading the extract on the SPE column, the cartridge was conditioned consecutively with 3 × 1 ml MeOH (LC-MS grade) and 3 × 1 ml water (LC-MS grade). After loading the extracts (1 ml), the cartridges were dried under vacuum and the analytes were eluted with 1 ml of a mixture of MeOH/ACN (1:1, v/v) containing 2% formic acid. The eluate was evaporated to dryness under a gentle stream of nitrogen, and the concentrated residue was redissolved in 1 ml MeOH. Efficiencies of SPE for metformin and guanylurea were calculated by comparing their CE-MS peak areas from the spiked fish tissue before and after the SPE procedure.

## Results and discussion

### Method development and optimization

#### Choice of the background electrolyte

With the low metformin concentrations present in environmental samples and the complex matrices, analytical methods with sufficient selectivity and sensitivity are required, not yet presented except for CE-ECL (LOD 0.3 μg/l) for urine samples [38] and CE-MS for serum samples (LOD 2.1 μg/l) [27]. With its high charge already at neutral pH, metformin is well amenable to CE as visible from the broad pH range used for its analysis which ranged from 2.5 [25] to 10 [39]. However, CE methods published so far mostly used background electrolytes incompatible with MS, e.g., phosphate buffers [23, 25, 29, 38]. Exceptions are the non-aqueous BGE made of 5 mM NH_4_OAc in MeCN + 5% HOAc which was published by Lai and Feng [26] for CE-UV analysis of metformin in human plasma and the aqueous BGE consisting of 50 mM formic acid for CE-MS analysis of metformin in human serum.

**Fig. 1:**
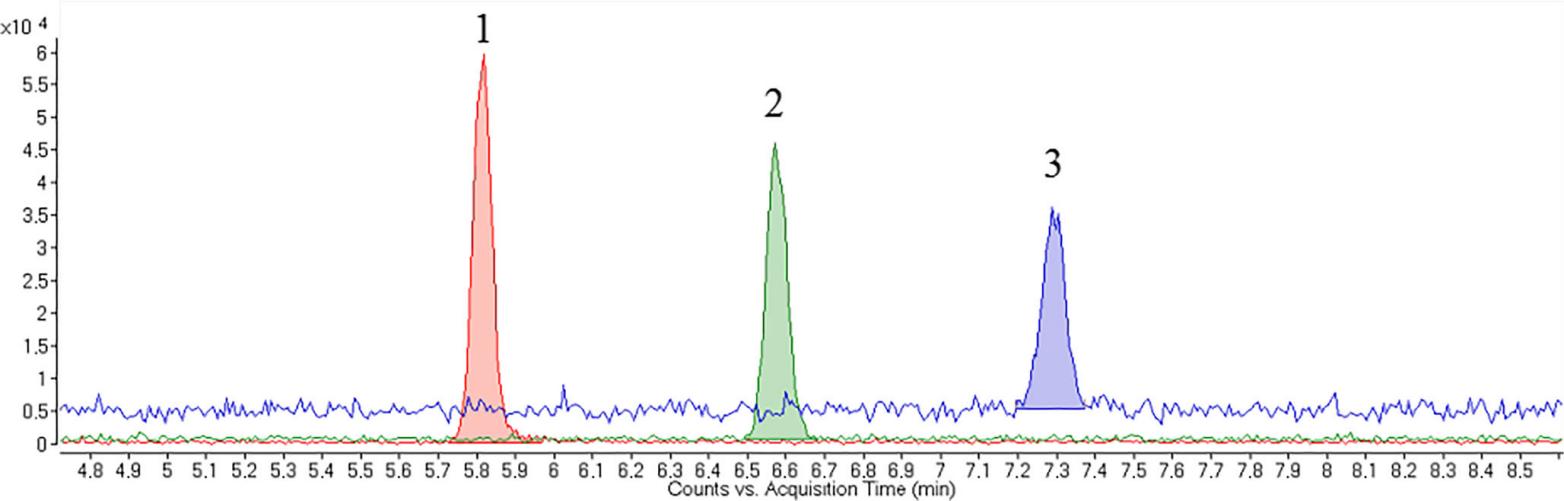
Extracted ion electropherograms of metformin m/z 130.1087 ± 0.001 in a methanolic standard solution (*c* = 100 nM) with varying NH_4_OAc concentrations in the BGE. (1) 25 mM NH_4_OAc in MeOH:HOAc (97:3). (2) 50 mM NH_4_OAc in MeOH:HOAc (97:3). (3) 100 mM NH_4_OAc in MeOH:HOAc (97:3). Separation conditions: +30 kV separation voltage, 80 cm capillary length, and injection of 100 mbar/10 s

In this study, first, common MS-compatible BGEs were screened for the analysis of metformin and guanylurea. Comparing results obtained with an aqueous BGE with formic acid, non-aqueous separation media made of ACN and MeOH revealed better peak shapes as well as higher detection sensitivity (see ESM Fig. S1), similar to the results by Lai and Feng [26] for CE-UV. We observed adsorption effects associated with an aqueous BGE, resulting in a lowered precision (see ESM Fig. S1). To prevent adsorption, we also tested capillaries coated with polyvinyl alcohol and *N*-acryloylamido-ethoxyethanol [40], but the precision as well as the performance were lower compared to the non-aqueous method in bare fused-silica capillaries. Due to frequent current breakdowns with ACN, MeOH was preferred in our study as main solvent. We did not consider other solvents. As a first step, different amounts of NH_4_OAc (25 mmol/l, 50 mmol/l, 75 mmol/l, and 100 mmol/l) in a mixture of MeOH and acetic acid (97:3) were tested. In previous studies, NH_4_OAc was observed to induce selectivity changes due to a separation mechanism based on ion pairing and heteroconjugation [41]. Figure 1 illustrates the dependence of the separation of metformin on the content of NH_4_OAc in the BGE. With increasing NH_4_OAc concentration, migration times increased (by changes in electrophoretic and electroosmotic mobilities), whereas the signal-to-noise ratio for metformin decreased. This can be explained by the effect of longitudinal diffusion producing broader peaks with reduced signal height and possible quenching effects by the higher salt load. At a concentration of 75 mM NH_4_OAc, no peak was visible for metformin, presumably because strong quenching effects were present. This indicates that the ion-pairing equilibrium between the free analyte and acetate versus their ion pair is strongly shifted to the ion pairs. For guanylurea, the results were similar (see Fig. S2 in the ESM). For both analytes, a salt concentration of 25 mM NH_4_OAc in the BGE led to the best signal-to-noise ratios.

**Fig. 2:**
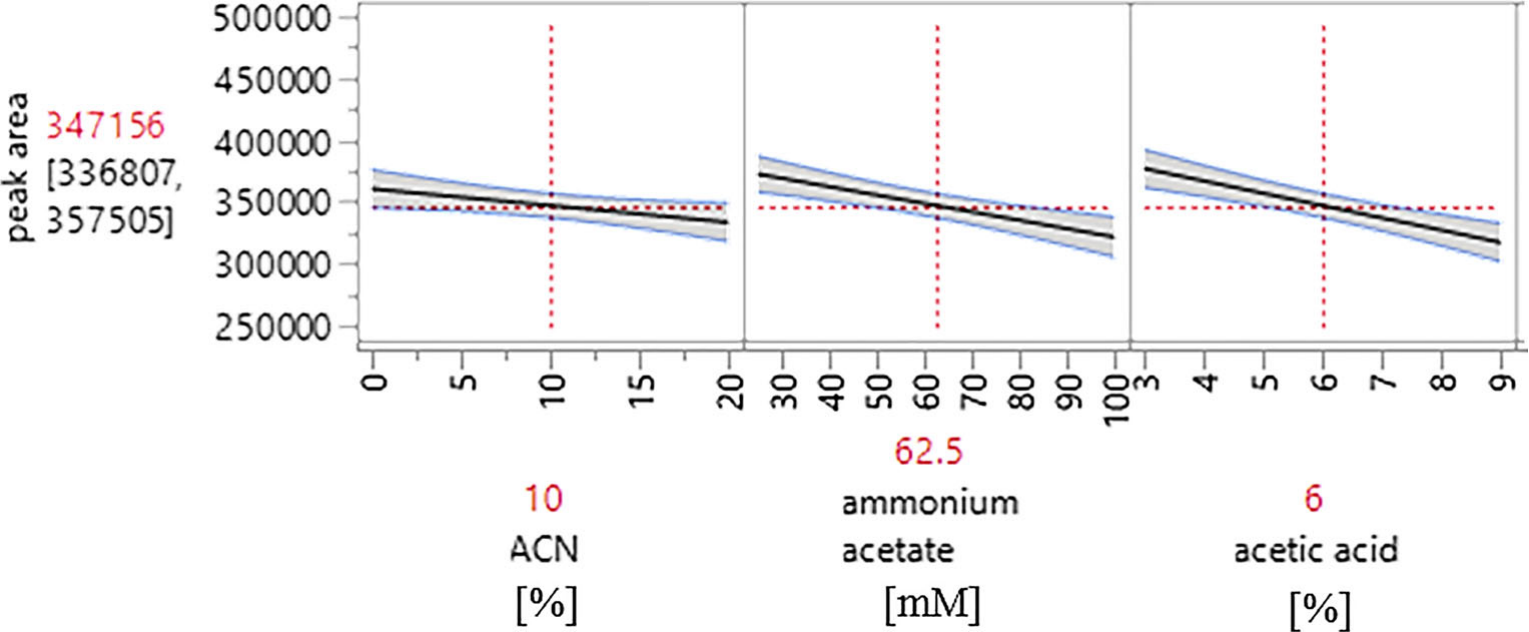
DOE results for metformin plotting the signal area versus the parameters ACN content in %, NH_4_OAc concentration in mmol/l, and HOAc content in %. Mean values are shown in red. Details of the DOE are given in the ESM

In order to determine the influence of all parameters of the BGE, namely the contents of ACN, NH_4_OAc, and HOAc in MeOH, and to understand their two- and three-factor interactions on the peak area as a measure for the detection sensitivity, a design of experiment (DOE) (2^3^ full factorial design, 11 measurements, *n* = 3) (see ESM Tables S1 and S2) was carried out. The results for metformin are summarized graphically in Fig. 2, which shows that the detection sensitivity is influenced by all parameters. According to the DOE, ideal separation conditions are as follows: no addition of ACN, a low NH_4_OAc concentration (25 mM), and a low HOAc content (3%). Proofs for significance by means of JMP software (version 13.0.0; SAS Institute, Böblingen; Germany) showed the following results (Table 2): the HOAc content and the NH_4_OAc concentration as well as interacting effects of the parameters ACN content and NH_4_OAc concentration were significant (*p* < 0.01) for the detection sensitivity of metformin. For NH_4_OAc concentrations up to 80 mM, the ACN content affects the peak area, but this is no longer relevant above 80 mM NH_4_OAc (see ESM Fig. S4). For guanylurea, similar results were obtained (see Table S3 and Fig. S3 in the ESM). The final method (see figure legends) provided a very good separation efficiency and selectivity for both compounds.

**Table 2:** Parameters of the DOE and *p* values for the peak areas of the CE-MS method for metformin

Process variable	*p* value signal area
HOAc	0.00003
NH_4_OAc	0.00016
ACN × NH_4_OAc	0.00780
ACN	0.02904
ACN × HOAc	0.04936
NH_4_OAc × HOAc	0.43350

Details of the DOE parameters and parameter ranges are given in Tables S1 and S2 in the ESM

As in our study, Lai and Feng [26] observed a higher separation efficiency with the non-aqueous BGE compared to an aqueous BGE. Although they used UV detection instead of MS detection, a LOD of 12 μg/l was reached for plasma samples, due to using electrokinetic injection for 36 s. In addition, the observed matrix effects were low at the high plasma concentrations (mg/l) of metformin.

### Method adaption for the analysis of fish extracts

**Fig. 3:**
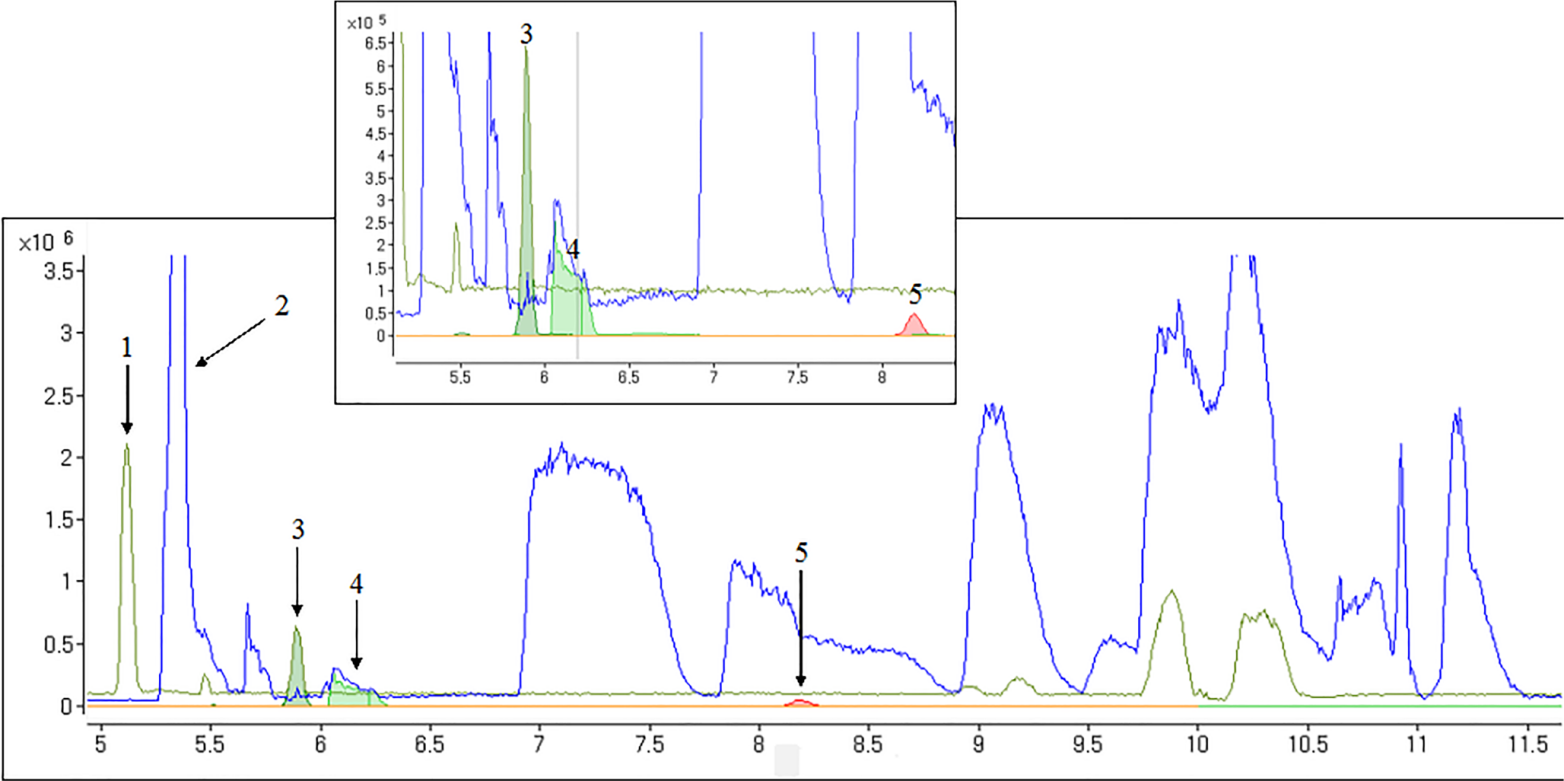
Base peak electropherogram m/z 100–300 and close-up of mass traces from (1) a methanolic zebrafish extract and (2) a methanolic brown trout extract. Extracted ion electropherograms of metformin m/z 130.1087 ± 0.001 of (3) a methanolic zebrafish extract, (4) a methanolic brown trout extract, and (5) extracted ion electropherogram of guanylurea m/z 103.0614 ± 0.001 of a methanolic zebrafish extract. Separation conditions: + 30 kV separation voltage, 80 cm capillary length, injection of 100 mbar/10 s, and BGE with 25 mM NH_4_OAc in MeOH:HOAc (97:3)

In order to assess the potential of the developed method for analyzing metformin and guanylurea in biological matrices, spiked fish samples (zebrafish embryos and brown trout) were analyzed. Figure 3 provides CE-MS electropherograms obtained for methanolic extracts of zebrafish. After spiking with metformin and guanylurea at a concentration of 1 μmol/l, samples were directly injected for CE-MS analysis. No interference by comigrating matrix components was visible comparing the extracted ion electropherogram with base peak electropherogram traces (Fig. 3). Clearly, the developed method proved highly selective for metformin and guanylurea in the zebrafish matrix.

**Fig. 4:**
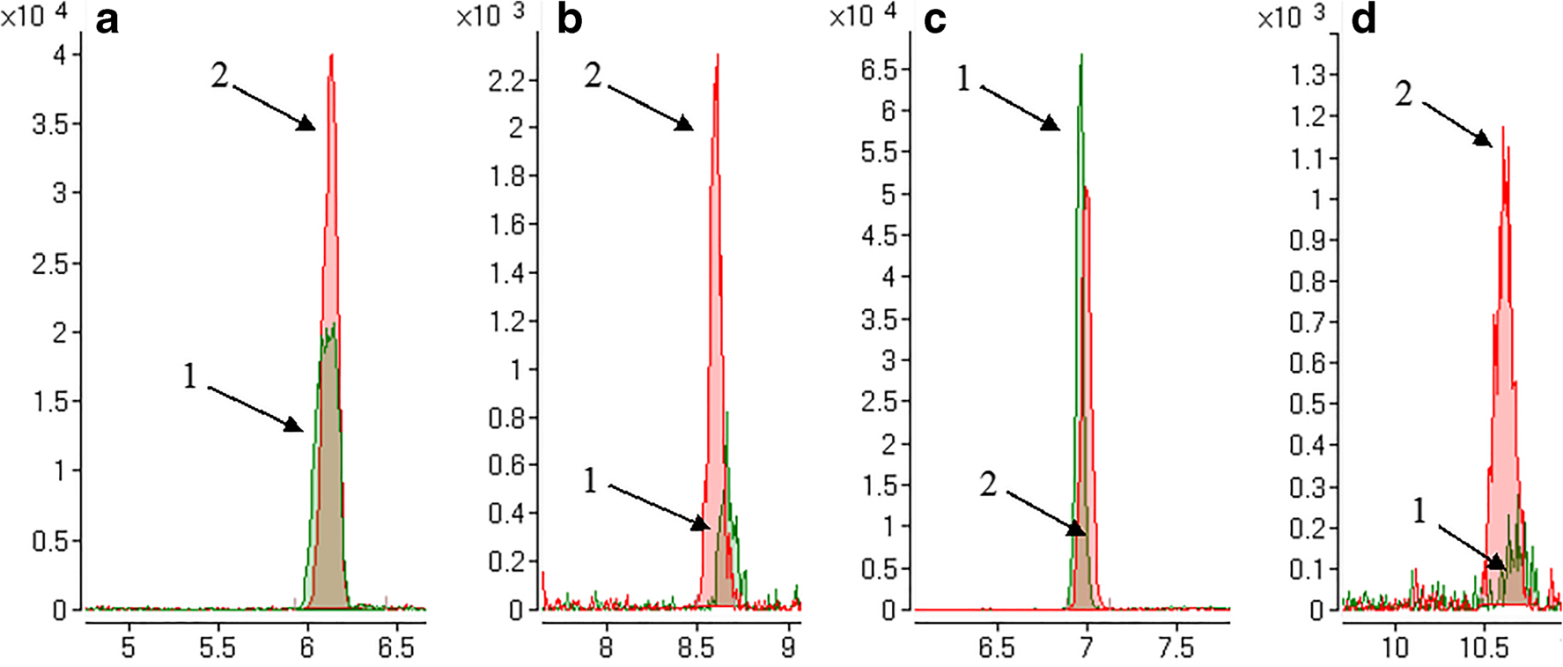
CE-MS analysis using a BGE solvent of MeOH:HOAc (97:3). (**a**) Extracted ion electropherogram of metformin recorded in a BGE with 25 mM NH_4_OAc. (**b**) Extracted ion electropherogram of guanylurea m/z 103.0614 ± 0.001, recorded in a BGE with 25 mM NH_4_OAc. (**c**) Extracted ion electropherogram of metformin m/z 130.1087 ± 0.001 recorded in a BGE with 100 mM NH_4_OAc. (**d**) Extracted ion electropherogram of guanylurea recorded in a BGE with 100 mM NH_4_OAc. EIC with m/z metformin = 130.1087 ± 0.001; m/z guanylurea = 103.0614 ± 0.001; mass traces from (1) a methanolic brown trout extract and (2) an SPE-purified brown trout extract; for further experimental separation conditions, see Fig. 3

In contrast, the matrix present in brown trout extracts was very complex, as visible from the base peak electropherograms obtained for both methanolic extracts in Fig. 3. Various signals of high intensity resulting from salts and endogenous, presumably amine-based compounds are visible. The comparison of the electropherograms revealed ion suppression for metformin in brown trout extracts, caused by comigrating matrix components (Fig. 3). To increase the selectivity and the matrix tolerance of the method, purification by SPE using a weak cation exchange mixed mode phase was tested. CE-MS results of SPE extracts were compared with those of a methanolic standard (Fig. 4a, b): For the methanolic extraction, not only the metformin signal but also the signal of guanylurea suffer from ion suppression caused by comigrating matrix components. After the clean-up, no interference by comigrating matrix components was detectable for both compounds, corroborated by comparison to data obtained using a deuterated standard for metformin (details not shown). Note that the SPE extract was preconcentrated a factor of 2 compared to the methanolic extract.

Beside sample preparation, selectivity may also be enhanced by optimizing the BGE. In case of non-aqueous CE, the ion-pairing constant can easily be influenced by the concentration of the BGE coion: Posch et al. [41] showed that the amount of ammonia (here, in the form of NH_4_OAc salt) in the non-aqueous BGE had very strong effects on the separation selectivity. Thus, the concentration of NH_4_OAc was increased from 25 to 100 mM to account for matrix effects. At 100 mM NH_4_OAc, the metformin signal was less affected by matrix components in both the methanolic and SPE extracts (Fig. 4c), but the improvement for metformin was particularly strong in case of the methanolic extract. Thus, as SPE has drawbacks with regard to manual effort and costs, further analyses of metformin in samples of brown trout were conducted after simple methanolic extraction using the adapted BGE of 100 mM NH_4_OAc, which provided somewhat lower separation efficiency and higher LODs for methanolic standards (see Fig. 1). Additionally, we tested different amounts of acetic acid (6% and 9%) in the BGE, but this did not lower ion suppression effects. Also, the polyimide layer was negatively affected by higher concentrations of acetic acid.

The electropherograms for guanylurea document a strong ion suppression in case of the methanolic extract, but to a significantly lower extent for the extracts purified via SPE (Fig. 4d). In contrast to metformin analysis, an increase of the NH_4_OAc concentration to 100 mM did not minimize signal suppression. A precise signal extraction of guanylurea from comigrating matrix components appeared to be impossible without a different sample preparation; i.e., the analysis of guanylurea in brown trout tissues required the use of SPE-purified extracts. Finally, we used a SPE clean-up and a BGE composed of 25 mM NH_4_OAc in MeOH:HOAc (97:3) to quantify guanylurea in brown trout.

## Figures of merit

### Linear range and limit of detection

The linear range of the peak area was tested for analytes dissolved in MeOH/BGE 90:10 (v/v), zebrafish, and brown trout fish matrix by spiking with metformin or guanylurea at five concentrations and plotting the peak area versus concentration. Each sample was consecutively injected three times. The calibration curves displayed good linearity with correlation coefficients (*R*) in the range of 0.959–0.998 (see Table 3). The LOD and the limit of quantitation (LOQ) were calculated via calibration curve for methanolic standards and fish extracts (zebrafish and brown trout) according to the German National Standard DIN 32645 [42] using calibrator concentrations of 0.1–40 μg/l for metformin and 5–70 μg/l for guanylurea and verified experimentally (see Table 3). The developed CE-MS method was able to detect both compounds in methanolic standard solution, zebrafish, and brown trout down to the lower μg/l range. For methanolic standards, a detection limit of 0.5 μg/l was obtained for metformin and 2 μg/l for guanylurea. As can be expected, the LOD values in fish matrix were higher for both compounds (see Table 3). Especially, the LOD of guanylurea in brown trout, which was 27 μg/l, was much higher compared to the LOD for the methanolic standard due to increased matrix effects. The LODs obtained for CE-MS are higher compared to those for analytical methods based on HILIC-MS, where LODs for both compounds in the low ng/l range could be reached for wastewater and surface water samples [43]. However, the results obtained in our study show that the LODs are sufficient for screening fish samples from exposure experiments. The developed method was compared with a CE-MS method for the analysis of metformin in human serum by Znaleziona et al. [27]. In this study, the sample preparation was also simple, as it was based on protein precipitation with ACN without any further clean-up. The LOD of metformin in blood serum was 2.14 μg/l, which is very similar to the LODs established for zebrafish embryos (4 μg/l) and brown trout (3 μg/l).

**Table 3:** Figures of merit for metformin and guanylurea determined in different matrices with their corresponding LOD, linear range, linear regression coefficient (in matrix), and matrix effect

Compound	Matrix	LOD in μg/l	LOQ in μg/l	LOD in ng/g	LOQ in ng/g	Linear range in μg/l	Sensitivity in counts × l/μg^a^	*R*^2^	Matrix effect in %^b^
Metformin	MeOH	0.5	1.4	–	–	0.1, 0.5, 1, 5, 10, 20, 30, 40	7089.9^c^	0.998	–
							6447.8^d^		
	Zebrafish embryos	4	12.2	–	–		7763.5^c^	0.967	19 ± 6^c^
	Brown trout larvae	3	9.3	15	23		5252.7^d^	0.978	− 21 ± 8^d^
Guanylurea	MeOH	2	5.6	–	–	5, 10, 20, 30, 40, 50, 60, 70	718.8^c^	0.993	–
							487.7^d^		
	Zebrafish embryos	5	15	–	–		757.5^c^	0.959	23 ± 11^c^
	Brown trout larvae	27	81	34	101		250.8^d^	0.961	− 52 ± 3^d^

For brown trout larvae, LOD and LOQ are also given in ng/g tissue homogenate

^a^Sensitivity is specified as the slope of the calibration curve

^b^Data are displayed as arithmetic means ± standard deviations

^c^BGE made of 25 mM NH_4_OAC in MeOH:HOAc (97:3)

^d^BGE made of 100 mM NH_4_OAC in MeOH:HOAc (97:3)

### Precision

Intraday precision was determined for three different concentrations of methanolic standard solutions (lower limit of quantification (LLOQ, 10 nM), mid-range concentration (100 nM), and high concentration (1 μM)), each injected six times (*n* = 6) consecutively. For metformin and guanylurea, an increased precision was observed at higher concentrations (above LLOQ): The precision of the peak area of metformin was 6% RSD for the LLOQ, 2% for the mid-range concentration, and 4% for the high concentration level. For guanylurea, the average RSD values were slightly higher with 14% for the LLOQ, 9% for the mid-range concentration, and 8% for the high concentration, which is also due to the higher LOD of guanylurea compared to metformin. Overall, all results for the intraday precision complied with the regulatory guidelines on bioanalytical method validation [44] stating that the intraday precision for the peak area of LLOQ should not exceed 15%, while the mid-range concentration and high concentration level should exhibit a precision < 20%. Znaleziona et al. [27] determined the intraday precision of the peak area for three concentration levels of metformin to range from 0.7 to 2.1%, which is in the same order of magnitude as in our study.

### Extraction efficiency

The efficiency of the extraction methods for zebrafish and brown trout was evaluated by means of recovery studies using spiked fish tissue. Several aliquots of homogenized fish samples were spiked (*n* = 3) with standard solutions (metformin-d6 and guanylurea). Recoveries for metformin and guanylurea in zebrafish were ≥ 95%. The extraction efficiency for the methanolic extraction of metformin in brown trout was 87% and thus slightly lower than for zebrafish. For SPE, the recovery was further reduced to 75% for metformin and 84% for guanylurea, which is most likely due to the loss of analyte during the several steps of SPE.

Scheurer et al. [9] applied SPE for the determination of metformin and guanylurea in aqueous environmental samples. With the same stationary phase (weak cation exchanger, Strata-X-CW), they reached a recovery of > 90% for metformin and 65–83% for guanylurea (depending on the matrix). In our study, the extraction efficiency for metformin (75%) is therefore lower, but even without a clean-up via SPE, an extraction efficiency of 87% could be established for metformin in brown trout samples. Furthermore, in the study by Scheurer et al. [8, 9], water samples and not fish samples were extracted. For guanylurea, the recovery is in the same order of magnitude as in our study (84%).

### Quantification of matrix effects

A major problem for ESI detection, particularly with complex matrices such as fish tissues, is ion suppression or signal enhancement by the presence of coextracted matrix components. To evaluate the effect of the matrix on the analysis of target compounds in different fish tissues, peak areas of metformin and guanylurea in fish extracts spiked at 20 μg/l, 40 μg/l, 80 μg/l, and 100 μg/l for metformin and guanylurea were compared to those of the analytes in solvent (MeOH:BGE 90:10, v/v) at the same concentration. Each sample was consecutively injected three times. Matrix effects (MEs) were calculated via the formula ME (%) = ((*A* − *B*) / *B*) × 100% (where *A* and *B* are the average peak area (*n* = 3) of the analyte in solvent and matrix, respectively) as mean values from three analytical replicates for zebrafish and brown trout tissues [45]. The percentage of signal reduction or enhancement for the compounds is given in Table 3. The matrix effects identified demonstrate that accurate quantification of metformin and especially guanylurea in fish extracts is not possible using calibration standards in MeOH. Common approaches for dealing with matrix interference include spiking with a labeled internal standard (ideally stable isotope labeled), using the method of standard addition, or employing matrix-matched calibration standards. While quantification with an internal standard is probably the simplest approach for the compensation of matrix interference in analyses employing mass spectrometry, costs and limited availability of labeled standards can be problematic. For guanylurea, an isotopically labeled standard was not commercially available. Therefore, matrix-matched calibration had to be employed to account for matrix interferences. Quantification of metformin in fish samples was performed with deuterated metformin (metformin-d6). The standard addition method was not considered.

The sensitivity of the method for both analytes and different matrices was determined as the slope of the calibration curve. For metformin, the sensitivity for different matrices was in the order zebrafish matrix > MeOH > brown trout, whereas the limit of detections were best in the order MeOH < brown trout < zebrafish (see Table 3). This result correlated well with the observed matrix effects, which were positive in zebrafish samples (19.4 ± 5.8%) but negative in brown trout (− 21.3 ± 7.7%). The strong positive effect for zebrafish samples was due to a transient isotachophoresis (tITP), which originated from the high salt concentration present in zebrafish matrix [46, 47] (see Fig. S5 in the ESM). Also for guanylurea, a positive matrix effect was observed for zebrafish (22.7 ± 10.5%) but a negative matrix effect for brown trout (− 51.6 ± 3.2%) (see Table 3).

## Tissue residues in real samples

### Zebrafish embryos

Tissue residues for zebrafish embryos were calculated as g/l as a single embryo cannot be weighed accurately [48]. The tissue residues of metformin in zebrafish after exposure to 0.5 g/l were 3 mg/l, when calculating a volume of 440 nl per zebrafish [49].

### Brown trout larvae

Fish samples taken from all exposure concentrations were analyzed. For quantification of metformin in fish tissue, an internal standard (metformin-d6) was used. The results of the analysis showed a dose-dependent tissue residue concentration of metformin. Exposure temperature was shown to have an effect on the uptake of the drug in the tissue: especially for the highest exposure concentration of 1000 μg/l, the tissue concentration of the drug was nearly four times higher in fish exposed at 11 °C than at the lower temperature. The measured metformin tissue concentrations were in the range of 4 to 234 ng/g dry weight [12]. There are a few toxicological studies that examined the effects of metformin exposure on fish [49, 50], but the bioaccumulation of the compound has not been analyzed. de Solla et al. [51] determined the bioaccumulation of metformin and other micropollutants in the unionid mussel *Lasmigona costata* from a river receiving wastewater effluent (up to 6–7 ng/g (wet weight)) with a method based on LC-MS/MS, but no figures of merit were presented. From our results, a maximal bioconcentration factor (BCF) of 0.2 was calculated (as the ratio of the concentration in biota vs. concentration in medium). Since the samples of brown trout larvae used for chemical analysis only contained kidney, muscle, and head without gills, but not intestines or liver, it was not possible to make a statement on the overall distribution of metformin in the fish. Several studies with mice and humans demonstrated that the highest metformin concentrations can be found in the gastrointestinal tract, kidney, and liver [52, 53]. Therefore, it was assumed that the measured concentrations in brown trout tissue were dominated by metformin in the kidney. For comparison, a metformin bioaccumulation factor (BAF) of 0.66 was in unionid mussel *Lasmigona costata* exposed to river water receiving wastewater effluent [51]. The BAF is calculated in the same way as the BCF; however, BAF is used when both ingestion and direct contact lead to the uptake of a substance.

### Tissue microanalysis of juvenile brown trout

**Fig. 5:**
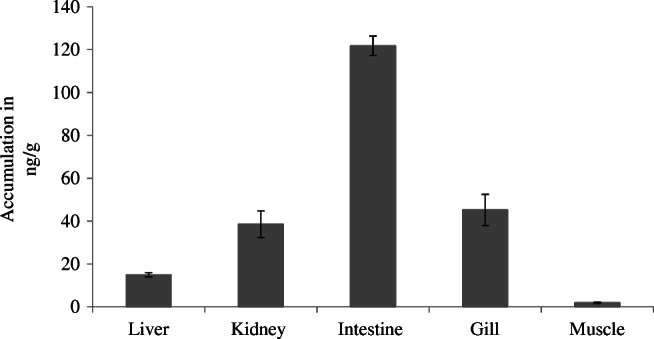
Accumulation of metformin in various biological tissues (liver, kidney, intestines, gill, and muscle) originating from five juvenile brown trout which were exposed to a metformin concentration of 1000 μg/l. The tissues of five juvenile brown trout were pooled, and three subsamples were generated per tissue type. Metformin was extracted following the protocol previously described for juvenile brown trout and analyzed by CE-MS. Metformin concentrations were calculated depending on the signal area of the internal standard. All data are shown as arithmetic mean ± standard deviation (*n* = 3)

To investigate the distribution of metformin in brown trout, various biological tissues of juvenile brown trouts (liver, kidney, intestines, gill, and muscle) originating from an exposure experiment with a concentration of 1000 μg/l were examined separately. For the analysis, tissue samples of five individuals were pooled and three subsamples were generated per tissue type. Due to the low sample requirement, CE-MS allowed a microanalysis of various biological tissues of juvenile brown trout separately. Metformin residue concentrations were quantified with the internal standard metformin-d6. As shown in Fig. 5, the intestines showed the highest accumulation of 122 ng/g metformin. Overall, the residue concentrations of metformin in brown trout followed the order intestines > gill > kidney > liver > muscle. The resulting BCFs in the different biological tissues ranged from 0.002 to 0.1. The results show that the highest metformin concentration was present in intestines, followed by gill, kidney, liver, and muscle. A possible explanation could be the high affinity of metformin to the negatively charged intestinal wall, resulting in an adsorption depot situated in the gastrointestinal tract [54, 55]. The concentration in the muscle tissue was only 2 ng/g, and therefore, it is much lower compared to the concentrations determined in the tissue of brown trout larvae. The main reasons are most likely the short exposure time of only 23 days, as well as the fact that only muscle tissue (without the kidney) was analyzed. This also supports the hypothesis that the concentrations in brown trout larvae are dominated by metformin in the kidney. The highest BCF of all exposure experiments with brown trout was determined to be 0.2, which demonstrates that metformin is not bioaccumulative according to REACH regulation [56]. Straub et al. [57] also showed that based on estimated BCFs ≤ 3.16, neither metformin nor guanylurea is expected to bioaccumulate in fish.

## Summary and conclusion

An analytical method using CE-MS for the selective, sensitive, fast, and cost-effective (running costs) analysis of metformin and guanylurea in fish samples and selected organs was presented in this study. The sample preparation consists of a simple methanolic extraction step for metformin and of a single SPE step for guanylurea, considerably simplifying sample preparation. The selectivity of the CE method could be optimized for different matrices by the adaptation of the BGE. The developed method was validated, and matrix effects were evaluated. Application of the method to the analysis of brown trout larvae samples originating from an exposure experiment with metformin revealed residue concentrations at the ng/g level. Microanalysis of selected organs was possible and showed that the highest metformin concentration was present in intestines, followed by gill, kidney, liver, and muscle. The results show that CE is well applicable to the analysis of very polar and charged micropollutants.

## Electronic supplementary material

ESM 1 (PDF 344 kb).
