# Supplementary material for: Development of a capillary electrophoresis–mass spectrometry method for the analysis of metformin and its transformation product guanylurea in biota
Source: Anal Bioanal Chem. 2020 Jun 22;412(20):4985–96. doi: 10.1007/s00216-020-02759-6 (PMC7334255; doi:10.1007/s00216-020-02759-6)
Supplement: Supplementary file 1 — (PDF 344 kb). [file 216_2020_2759_MOESM1_ESM.pdf]

Analytical and Bioanalytical Chemistry

Electronic Supplementary Material

Development of a capillary electrophoresis – mass spectrometry method for the analysis of metformin and its transformation product guanylurea in biota

Sarah Knoll, Stefanie Jacob, Susanna Mieck, Rita Triebskorn, Thomas Braunbeck, Carolin Huhn

Exposure of zebrafish embryos

Adult zebrafish (*Danio rerio*) used for breeding were maintained in the fish facilities of the Aquatic Ecology and Toxicology Group at the Centre for Organismal Studies (COS), University of Heidelberg (licensed under no. 35-9185.64/BH). The zebrafish were wild-type descendants of the “West-Aquarium” strain. For details on fish maintenance and breeding conditions, see Lammer et al. (2009). Embryos were staged according to Kimmel et al. (1995) and reared in crystallizing dishes at a maximal density of 50 embryos per dish in 200 ml artificial water at a temperature of 26 ± 1 °C, a pH of 7.75, and a 16:8 h light-dark regime. The culture medium was changed daily.

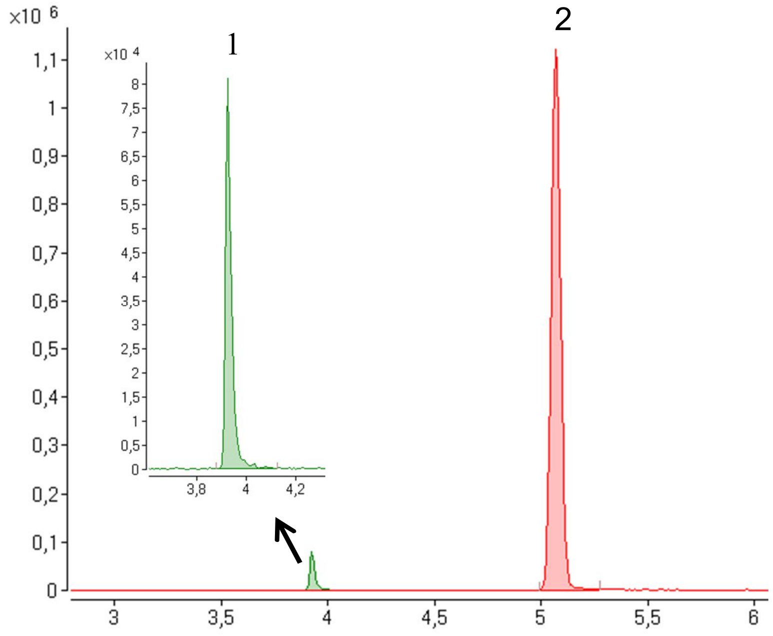

Fig. S1 Extracted ion electropherograms (intensity (counts) vs. migration time) of metformin m/z 130.1087 ± 0.001 (inte in a methanolic standard solution with varying BGE: (1) 175 mM formic acid, pH 2.8; (2) 50 mM NH_4OAc in MeOH:HOAc (97:3); Separation conditions: +30 kV; 80 cm capillary length; injection: 100 mbar 10 s

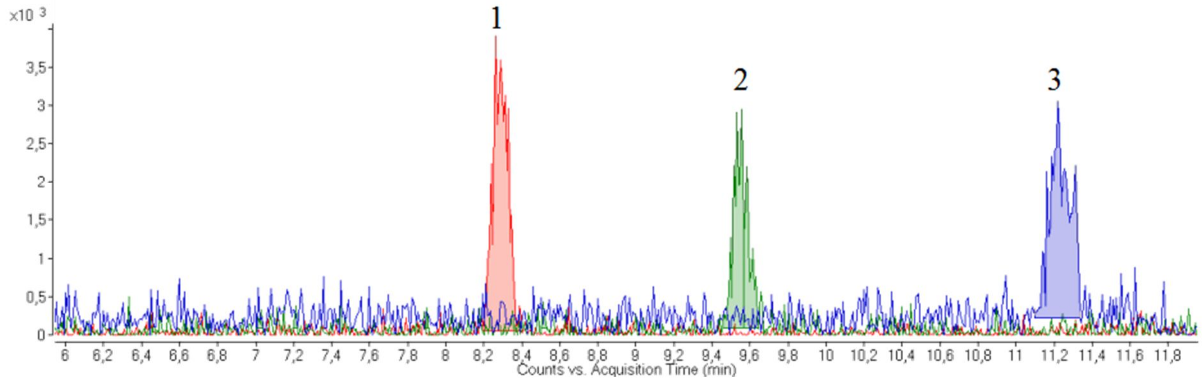

Fig. S2 Extracted ion electropherograms of guanylurea m/z 103.0614 ± 0.001 in a methanolic standard solution (c = 100 nM) with varying NH_4OAc concentration in the BGE: (1) 25 mM NH_4OAc in MeOH:HOAc (97:3); (2) 50 mM NH_4OAc in MeOH:HOAc (97:3), (3) 100 mM NH_4OAc in MeOH:HOAc (97:3); Separation conditions: +30 kV; 80 cm capillary length; injection: 100 mbar 10 s

Table S1 Experimental design of the DOE; ammonium acetate was dissolved in methanol

factors	levels low (-)/high (+)	standard condition (0)
MeCN	0 %/20 %	10 %
NH_4OAc	25 mM/100 mM	50 mM
HOAc	3 %/9 %	6 %

Table S2 Matrix design of the DOE (2^3 full factorial design); 0, -, + as given in Table S1

experiment number	MeCN	NH_4OAc	HOAc
1	-	-	-
2	+	-	-
3	-	+	-
4	+	+	-
5	-	-	+
6	+	-	+
7	-	+	+
8	+	+	+
9	0	0	0
10	0	0	0
11	0	0	0

Table S3 Parameters of the DOE and p-values for the sensitivity of the CE-MS method for guanylurea calculated for the signal area

Process variable	p-value signal area
ACN	0
NH_4OAc	0
HOAc	0.00736
NH_4OAc * HOAc	0.004489
ACN * NH_4OAc	0.13561
ACN * HOAc	0.34381

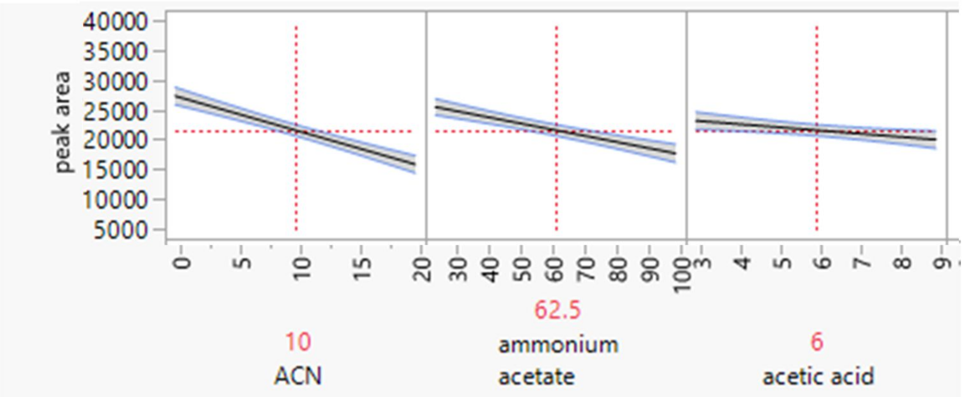

Fig. S3 DOE results for guanylurea plotting the signal area *vs*. the parameters ACN content in %, NH_4OAc concentration in mmol/l and HOAc content in %; mean values are shown in red. See also Tables S1 and S2

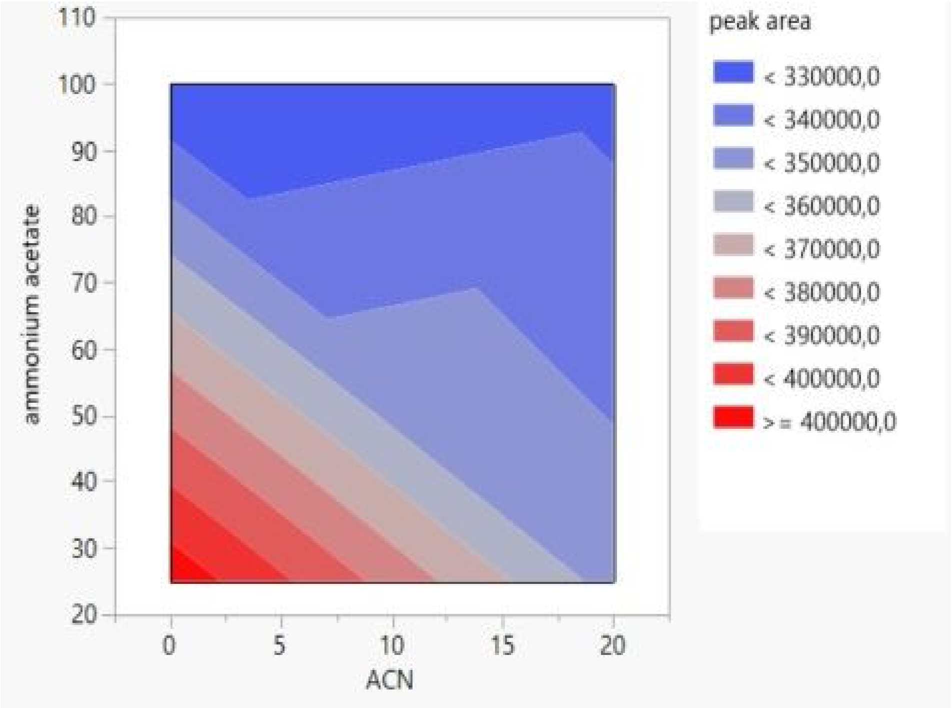

Fig. S4 Contour plot showing interacting effects of the parameters ACN content in % and NH_4OAc concentration in mmol/l on the peak area of metformin signals

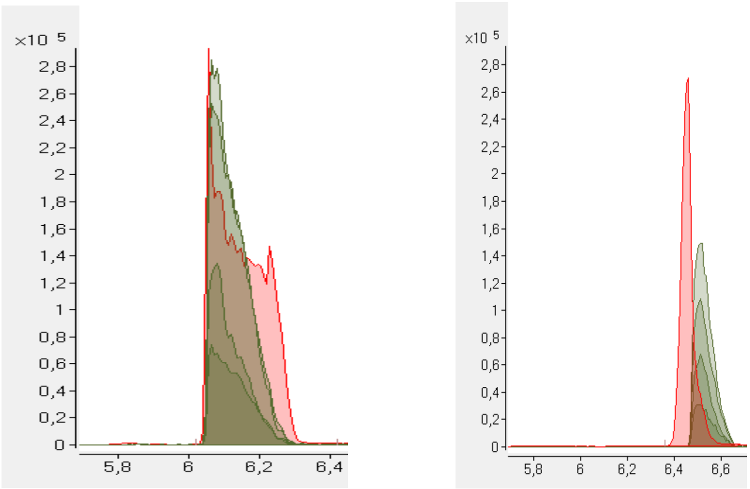

Fig. S5 Electropherograms demonstrating the impact of a transient ITP during the analysis of methanolic extracts with MeHO:AcOH and a) 50 mM NH_4OAc and extracts from brown trout. The metformin signal is shown in red, others stem from matrix components. Clearly, on the left side, comigration is visible, the broad sqaure-shaped signals indicate an ITP stack, not resolved upon detection or in the transition to CE. We asssume that inorganic ions are also present in this stack leading to a broad signal. On the right side, selectivity changes lead to the separation of metformin from matrix components. Either the transient mixed zone visible on the left, has evolved, or –ro us more likely- inorganic matrix ions als now also well separated from the matrix components, so that the transient ITP resolved befor detection
